# Chronic Kidney Disease Study in Diabetic Patients: Insights From Primary Care Units in Northern Portugal

**DOI:** 10.7759/cureus.61417

**Published:** 2024-05-31

**Authors:** Ricardo J Afonso, Maria Teixeira, Diana Murteira, Nilza Tavares, Magui Neto, Hugo A Gomes, Brenda G Jorge, Jéssica Tavares, Maria Santos, Cristiana Soares

**Affiliations:** 1 USF (Family Health Unit) Salvador Machado, Unidade Local de Saúde (ULS) Entre Douro e Vouga, Oliveira de Azeméis, PRT; 2 USF (Family Health Unit) Santo António, Unidade Local de Saúde (ULS) Barcelos/Esposende, Barcelos, PRT; 3 USF (Family Health Unit) Entre Margens, Unidade Local de Saúde (ULS) Entre Douro e Vouga, Pinheiro da Bemposta, PRT; 4 USF (Family Health Unit) Calâmbriga, Unidade Local de Saúde (ULS) Entre Douro e Vouga, Vale de Cambra, PRT

**Keywords:** chronic renal insufficiency, albuminuria, primary health care, diabetes mellitus, diabetic nephropathies

## Abstract

Introduction

Diabetes mellitus (DM) remains a primary cause of morbidity and mortality, leading to complications such as blindness, kidney failure, and lower limb amputations. Early detection of kidney damage, indicated by microalbuminuria (MA), is crucial for managing DM. Given the impact of these conditions, evaluating the prevalence of chronic kidney disease (CKD) in diabetic populations within primary healthcare is essential.

Methodology

This was a cross-sectional and observational study. Adults diagnosed with DM type 1 or 2*, *from five primary care units (PCUs) located in the North of Portugal, were included in this study. Descriptive and correlational statistics were performed using IBM SPSS Statistics for Windows, Version 28.0 (IBM Corp., Armonk, NY). Statistical significance was set to *P *< 0,05. Logistic regression models were created to identify the factors associated with CKD and DM*.*

Results

A sample of 357 diabetic patients was obtained, with 166 (46.5%) females. Of the sample, 250 (70.1%) were aged 65 or older, and the median known duration of DM was 9.36 years. Excess weight or obesity accounted for 79.8%, with a median body mass index of 28.73 kg/m^2^ and hypertension in 284 (79.6%). An estimated glomerular filtration rate (eGFR) less than 60 mL/min was present in 89 (24.9%) and an MA of 30 mg/dL or higher was present in 68 (19.0%). In total, 130 (36.4%) individuals exhibited eGFR and MA consistent with CKD. Among these, 25 (78.1%) had other identifiable causes of CKD besides DM, hypertension, overweight, or obesity. Binary logistic regression models were constructed to find a relationship between CKD with eGFR < 60 mL/min and MA. A statistically significant association was found between CKD with eGFR < 60 mL/minute and age (odds ratio [OR] = 1.150; *P *< 0.001), kidney stones (OR = 5.112; *P *= 0.003), absence of excess weight or obesity (OR = 0.267; *P *< 0.001). The use of GLP1 agonists showed statistical significance as a predictor (OR = 4.653; *P *= 0.042) of the presence of MA.

Discussion

The study investigates the impact of DM and its complications in the surveyed population. While most patients had controlled DM (284, 76.2%), prolonged disease duration correlated with poorer glycemic control, underscoring the need for more effective management strategies in advanced disease stages. Notably, a third of individuals with DM had CKD, with significant implications for therapeutic interventions and heightened risks of renal failure and cardiovascular morbidity. MA was a crucial marker for endothelial injury, with prevalence influenced by DM duration and medication type. However, in many cases, correct identification of CKD was lacking, suggesting under-recognition of renal deterioration in DM. While the study offers valuable insights, its limited sample size and geographic scope warrant cautious interpretation, emphasizing the need for broader, context-specific research to inform comprehensive healthcare strategies.

Conclusions

In conclusion, this study highlights the significant burden of CKD among diabetic patients, emphasizing the need for proactive screening, personalized management, and accurate diagnosis. Despite limitations, it underscores the importance of early detection and tailored interventions, advocating for improved diabetes care to mitigate renal complications on a broader scale.

## Introduction

Currently, diabetes mellitus (DM) has a substantial global impact. It is estimated that 10.5% of adults worldwide have DM, and among people over 65, this figure rises to one in five. Projections indicate that the total number of individuals with DM will increase to 643 million (one in nine adults) by 2030 and 784 million (one in eight adults) by 2045 [1].

In Portugal, the estimated prevalence of DM among individuals between 20 and 79 years is 14.1% (1.1 million cases). There's a notable correlation between age and DM prevalence, with over a quarter of individuals aged 60-79 having DM. Moreover, it's speculated that only 56% of affected individuals have been diagnosed [1].

Despite a significant decrease in the potential years of life lost due to DM over the last decade, this condition remains a primary cause of both morbidity and mortality. In 2021 alone, DM accounted for 6.7 million deaths globally and was responsible for 11.5% of healthcare expenditures [1].

Individuals with DM are susceptible to various complications. Across virtually all developed nations, DM is the primary cause of blindness, kidney failure, and lower limb amputations. DM prevalence in people with chronic renal insufficiency is notably high, reaching 28%, according to the annual report of the National Diabetes Observatory of the Portuguese Diabetology Society [1]. Therefore, implementing preventive strategies is crucial in managing this disease. Risk stratification and early detection of kidney damage are pivotal in guiding the treatment of individuals with DM.

Chronic kidney disease (CKD) is diagnosed based on a reduced glomerular filtration rate and/or the detection of elevated levels of urinary albumin excretion. Kidney damage can be identified through the presence of microalbuminuria (MA), an early indicator of damage [2]. MA is characterized by a moderate increase in albumin excretion in urine (ranging between 30 and 300 mg/day) [3].

MA can be assessed via 24-hour proteinuria (considered the reference method) or the albumin-creatinine ratio (ACR) obtained from a urine sample [2]. A recent study in Portugal revealed that using urine samples to detect MA demonstrated high sensitivity (>91%) in hypertensive individuals with or without DM, along with a high negative predictive value (>96%) compared to the 24-hour collection. This confirms the reliability and usefulness of this method in MA screening [4,5].

MA is currently regarded as a significant risk factor for cardiovascular disease and mortality in patients with DM and/or hypertension (HT), leading to generalized endothelial/vascular dysfunction, supported by several published studies [6,7]. According to data from the latest annual report of the Annual Observatory for Diabetes, 67.3% of diabetics had their MA analyzed in 2021, with 20.4% exhibiting values exceeding 30 mg/24 hours [1].

Considering the clinical heterogeneity of CKD and its impact on the progression and morbidity-mortality of specific populations, particularly in patients with DM, the present study was conducted to assess the prevalence and impact of this condition in a diabetic population managed in a primary healthcare setting.

## Materials and methods

Transverse and observational study, involving diabetic patients aged 18 years and above was conducted, across five primary care units (PCUs), located in the northern region of Portugal, encompassing both urban and rural areas. In January 2023, 5,036 patients with a diagnosis of DM were identified in MIM@UF, considering if International Classification of Primary Care, 2nd Edition (ICPC-2) code T89 or T90 was present. A representative sample of 357 diabetics was included in the study after random selection. To determine the representativeness of the sample, with a 95% confidence interval and a 5% margin of error, an online calculator, freely available, was used [8].

The data collection period spanned from January to June 2023, during which investigators gathered information from the medical records acquired from the electronic health record system SClinico. Multiple predictive variables were collected, including age (in years), gender, body mass index (BMI) (kg/m^2^), ACR (mg/g), estimated glomerular filtration rate (eGFR) (mL/min/1.73 m^2^), Kidney Disease: Improving Global Outcomes (KDIGO) cardiovascular risk stratification, HT control, DM control, known DM duration (in years), use of renin-angiotensin system inhibitors or antidiabetic medication, presence of adequate coding in CKD patients (ICPC-2 coding U88 and/or U99 present), and adequacy of antidiabetic treatment considering eGFR. Furthermore, the presence of other potential causes of CKD was determined (benign prostatic hyperplasia, kidney stones, overweight or obesity, and other causes). When only two or fewer cases were identified, they were grouped as *other causes* (polycystic kidney, pyelonephritis, glomerulonephritis, iatrogenic, etc.).

The presence of at least MA was defined as an ACR >= 30 mg/g in a spot urine sample. The eGFR was assessed using the modified Cockcroft-Gault formula. All eGFR values greater than 120 mL/min were adjusted to a maximum of 120 mL/min. DM control was defined based on HbA1c values at the last consultation. Patients with HbA1c values less than 6.0% and aged under 65 years, or HbA1c 6.0% to 7.0% in patients aged between 65 and 75 years old, or HbA1c below or equal to 7.5% in patients aged 75 years or older were considered controlled. HT control was defined as an office blood pressure (BP) value <140/90 mmHg or, in patients aged 80 years or older, <150/90 mmHg.

Data collection was performed by the investigators, and family medicine residents from the respective PCU, following protocol approval by the Ethics Committee for Health of the Northern Regional Health Administration with the reference number CE/2023/78. Statistical analysis was conducted using IBM SPSS Statistics for Windows, Version 28.0 (IBM Corp., Armonk, NY), with a statistical significance level of 5% (*P* < 0.05). For categorical variables, absolute and relative frequencies were determined. Continuous variables were characterized using measures of central tendency (median) and dispersion (25th and 75th percentiles) due to their non-normal distribution, assessed using the Kolmogorov-Smirnov test with Lilliefors correlation (*P *< 0.05).

To establish relationships between continuous variables with non-normal distributions, the investigators employed nonparametric tests such as the Mann-Whitney U test and the Kruskal-Wallis test for continuous variables, and chi-square tests or Fisher's exact tests for categorical variables. Values with *P *< 0.05 were considered statistically significant. Binary logistic regression modeling was utilized for multivariate analysis of the data.

## Results

A sample of 357 diabetic patients was obtained, with 166 (46.5%) being female, and a median age of 71.00 years (P25-P75 62.00-79.00). Women had a higher median age of 73 years (P25-P75 65.00-81.75; *P *< 0.001), while men had a median age of 69 years (P25-P75 59.00-78.00). The majority (250, 70.1%) were aged 65 years or older, and 132 (37%) were 75 years or older. Additionally, it is noteworthy that type 1 DM represented only 14 cases (3.9%). More detailed demographic data can be found in Table 1.

**Table 1:** Demographic characterization The data are represented as *n*, %, median (P25-P75), mean ± SD. PCU, primary care unit; USF, Family Health Unit

Demographic features	n	%
Totality of the sample	357	100.0
Male sex	191	53.5
Age groups (years)		
<40	6	1.7
40-64	101	28.3
65-74	118	33.1
>=75	132	37.0
Median age (P25-P75)	71.00 (62.00-79.00)	
MV ± SD	69.91 ± 12.40	
PCU		
USF Salvador Machado	150	39.2
USF La Salette	56	15.7
USF Santo António	55	15.4
USF Entre Margens	55	15.4
USF Calâmbriga	51	14.3

Excess weight or obesity accounted for 81.8% (*n* = 292) of comorbidities, with a median BMI of 28.73 kg/m^2^ (P25-P75 25.65-31.25), HT was present in 79.6% (*n* = 284) of cases. Among these, 76.2% (*n *= 216) had their BP under control. The median duration of DM was 9.36 years (P25-P75 6.00-17.50), with no statistically significant differences found between genders (*P *= 0.067), presence or absence of HT (*P *= 0.181), and presence or absence of excess weight or obesity (*P *= 0.630). However, type 1 DM had a longer duration of illness (*P *= 0.029); and an increase in years of illness corresponded to poorer DM control (*P *< 0.001). Of all patients, 76.2% (*n *= 272) had their DM under control. A statistically significant difference (*P *< 0.001) was found in the median duration of illness between insulin-medicated and non-insulin-medicated groups. More detailed clinical data are given in Table 2.

**Table 2:** Clinical characterization. Data are represented as *n*, %, median (P25-P75), mean ± SD. eGFR, estimated glomerular filtration rate; GLP1-a, glucagon-like peptide-1 agonist; SGLT2-i, sodium-glucose co-transporter-2; DPP4-i, dipeptidyl peptidase 4 inhibitors; ACEi, angiotensin-converting enzyme inhibitor; ARB, angiotensin II receptor blocker; SD, standard deviation

Clinical features	Median (P25-P75)	MV ± SD
Body mass index (kg/m^2^)	28.73 (25.65-31.25)	28.73 ± 4.71
Glycated hemoglobin, HbA1c (%)	6.80 (6.20-7.50)	6.98 ± 0.202
Diabetes duration (years)	9.36 (6.0-17.50)	13.15 ± 1.16
Creatinine (mg/dL)	0.85 (0.73-1.01)	0.95 ± 0.46
eGFR (mL/min)	79.30 (59.90-103.05)	79.88 ± 27.42
Diabetes mellitus classification	n	%
Type 1	14	3.9
Type 2	342	95.8
Diabetes mellitus control		
Controlled	272	76.2
Uncontrolled	85	23.8
Diabetes mellitus treatment		
Nonpharmacological treatment	12	3.4
Biguanide	285	79.8
Sulphonylurea	64	17.9
GLP1-a	15	4.2
SGLT2-i	85	23.8
DPP4-i	108	30.3
Glitazone	11	3.1
Insulin	59	16.5
Antidiabetic pharmacological associations		
Oral antidiabetics monotherapy	148	41.5
2-3 oral antidiabetics	126	35.3
>=4 oral antidiabetics	12	3.4
Insulin	38	10.6
ACEi or ARB medication (*n* = 283)		
Without ACEi or ARB	35	12.4
ACEi	154	54.4
ARB	94	33.2

Out of the total sample, 130 (36.4%) exhibited eGFR and/or ACR consistent with CKD. Among these, according to the KDIGO cardiovascular risk stratification, 80 (22.4%) presented a moderate risk and 50 (14.0%) presented a high or very high risk. The coding of CKD was correct in 23.1% (32/130) of CKD patients. Among these, 25 (78.1%) had other identifiable causes of CKD besides DM, HT, overweight, or obesity (Table 3).

**Table 3:** Chronic kidney disease (CKD) risk factors, classification, and risk stratification. Data are represented as *n* and %. eGFR, estimated glomerular filtration rate

Identifiable risk factors for CKD (other than DM)	n	%
Obesity or overweight	292	81.8
Arterial hypertension	284	79.6
Nephrolithiasis	29	8.1
Benign prostatic hyperplasia	29	8.1
Nonidentifiable risk factors for CKD	38	10.6
CKD stage according to eGFR (mL/min per 1.73 m^2^)		
G1 (>=90)	133	37.3
G2 (60-89)	135	37.8
G3a (45-59)	49	14.0
G3b (30-44)	29	8.1
G4 (15-29)	10	2.8
G5 (<15)	0	0.0
Persistent albuminuria KDIGO categories (mg/g)		
A1 - Normal to mildly increased (<30)	289	81.0
A2 - Moderately increased (30-300)	60	16.8
A3 - Severely increased (>300)	8	2.2
CKD KDIGO risk		
Low risk	227	63.6
Moderate risk	80	22.4
High risk	27	7.6
Very high risk	23	6.4

Regarding CKD, 133 (37.3%) had an eGFR equal to or greater than 90 mL/min, 135 (37.8%) had an eGFR between 60 and 89 mL/min, and 89 (24.9%) had an eGFR <60 mL/min. No statistically significant difference was found between the groups with eGFR equal to or greater than 60 mL/min and <60 mL/min regarding DM control (*P *= 0.274). The median age was higher, at 82.0 years (P25-P75 75.0-86.0), for those with an eGFR less than 60 mL/min, compared to the others, who had a median age of 68.0 years (P25-P75 59.3-68.0). The same trend was observed between known DM duration and eGFR greater than or equal to 60 mL/min and less than 60 mL/min, respectively, 10.0 years (P25-P75 5.0-16.0) and 16.0 years (P25-P75 9.0-23.0). It was also noted that an eGFR less than 60 mL/min was more frequent in women (*P *= 0.018), individuals without excess weight or obesity (*P *< 0.001), with nephrolithiasis (*P *= 0.034), and individuals medicated with inhibitors of dipeptidyl peptidase 4 (DPP4-i) (*P *= 0.007) or insulin (*P *= 0.038). Conversely, patients medicated with biguanides more frequently had an eGFR equal to or greater than 60 mL/min (*P *= 0.002) (Table 4).

**Table 4:** Association between estimated glomerular filtration rate and other variables. Data are represented as *n*, %, and median (P25-P75). *P*-value < 0.05 is considered significant. ^‡^Mann-Whitney U test. ^*^Chi-square test. ^†^Fisher’s exact test. eGFR, estimated glomerular filtration rate; CKD, chronic kidney disease; DM, diabetes mellitus; DPP4-i, dipeptidyl peptidase 4 inhibitor; GLP1-a, glucagon-like peptide-1 agonist; SGLT2-i, sodium-glucose co-transporter-2 inhibitor

Variable	eGFR (mL/min/1.73 m^2^)				
	eGFR >= 60		eGFR < 60		
	n	Median (P25-P75)	n	Median (P25-P75)	*P*-value
Age (years)	268	68.0 (59.3-68.0)	89	82.0 (75.0-86.0)	<0.001^‡^
Body mass index (kg/m^2^)	268	28.4 (26.2-32.0)	89	26.7 (23.7-30.2)	<0.001^‡^
Diabetes duration (years)	268	10.0 (5.0-16.0)	89	16.0 (9.0-23.0)	<0.001^‡^
Last glycated hemoglobin, HbA1c (%)	268	6.8 (6.1-7.5)	89	6.8 (6.2-7.8)	0.212^‡^
Creatinine (mg/dL)	268	0.8 (0.7-0.9)	89	1.2 (0.9-1.5)	<0.001^‡^
Gender	n	%	n	%	
Male	153	42.9	38	10.6	0.018*
Female	115	32.2	51	14.3	
DM classification					
Type 1	11	3.1	3	0.8	1.000†
Type 2	257	72.0	86	24.1	
DM control					
Controlled	208	58.3	64	17.9	0.274*
Uncontrolled	60	16.8	25	7.0	
Identifiable risk factors for CKD (other than DM)					
Obesity or overweight	228	63.9	57	16.0	<0.001*
Arterial hypertension	207	58.0	77	21.6	0.060*
Nephrolithiasis	17	4.8	12	3.4	0.034*
Benign prostatic hyperplasia	22	6.2	7	2.0	0.918*
Nonidentifiable risk factors for CKD	22	6.2	16	4.5	0.010*
DM treatment					
Biguanide	224	83.6	61	17.1	0.002*
Sulphonylurea	43	12.0	21	5.9	0.108*
GLP1-a	10	23.5	5	1.4	0.541†
SGLT2-i	66	18.5	19	5.3	0.529*
DPP4-i	52	14.6	37	10.4	0.007*
Glitazone	9	2.5	2	0.6	0.738†
Insulin	38	10.6	21	5.9	0.038*

Regarding ACR >= 30 mg/g, it was present in 68 (19.0%) individuals in the study sample. When comparing the groups with and without albuminuria, statistically significant differences were found. Albuminuria was more common with a longer duration since the diagnosis of DM (*P *= 0.009) and in individuals medicated with glucagon-like peptide-1 agonist (GLP1-a) (*P *= 0.046). Conversely, it was less frequent in patients medicated with biguanides (*P *= 0.035). More detailed information is given in Table 5.

**Table 5:** Association between albumin-creatinine ratio and other variables. Data are represented as *n*, %, and median (P25-P75). *P*-value < 0.05 is considered significant. ^‡^Mann-Whitney U test. ^*^Chi-squared test. ^†^Fisher’s exact test. ACR, albumin-creatinine ratio; CKD, chronic kidney disease; DM, diabetes mellitus; DPP4-i, dipeptidyl peptidase 4 inhibitor; GLP1-a, glucagon-like peptide-1 agonist; SGLT2-i, sodium-glucose co-transporter-2 inhibitor

Variable	ACR (mg/g)				
	ACR < 30		ACR >= 30		*P*-value
	n	Median (P25-P75)	n	Median (P25-P75)	
Age (years)	289	70.0 (61.0-79.0)	68	73.5 (64.5-81.5)	<0.126^‡^
Body mass index (kg/m^2^)	289	27.9 (25.9-31.1)	68	28.7 (24.6-31.9)	<0.994^‡^
Diabetes duration (years)	289	11.0 (50.0-17.0)	68	14.5 (8.0-20.5)	<0.009^‡^
Last glycated hemoglobin, HbA1c (%)	289	6.7 (6.1-7.50)	68	7.1 (6.3-8.1)	0.042^‡^
Creatinine (mg/dL)	289	0.8 (0.7-1.0)	68	1.0 (0.8-1.3)	<0.001^‡^
Gender	n	%	n	%	
Male	153	42.9	38	10.6	0.662*
Female	136	38.1	30	8.4	
DM classification					
Type 1	11	3.1	3	0.8	0.735†
Type 2	278	77.9	65	18.2	
DM control					
Controlled	224	62.7	48	13.4	0.228*
Uncontrolled	65	18.2	20	5.6	
Identifiable risk factors for CKD (other than DM)					
Obesity or overweight	235	65.8	50	14.0	0.150*
Arterial hypertension	226	63.3	58	16.2	0.192*
Nephrolithiasis	22	6.2	7	2.0	0.472*
Benign prostatic hyperplasia	26	7.3	3	0.8	0.213*
Nonidentifiable risk factors for CKD	27	7.6	11	3.1	0.100*
DM treatment					
Biguanide	237	66.4	48	13.4	0.035*
Sulphonylurea	57	16.0	7	2.0	0.068*
GLP1-a	9	2.5	6	1.7	0.046†
SGLT2-i	68	19.0	17	4.8	0.798*
DPP4-i	85	23.8	23	6.4	0.476*
Glitazone	8	2.2	3	0.8	0.445†
Insulin	43	12.0	16	4.5	0.084*

To neutralize the effect of confounding variables, adjusted binary logistic regression models were constructed.

Thus, the investigators tried to verify whether the variables gender, age, known DM duration, excess weight or obesity, kidney stones, other causes of CKD, use of biguanide, use of DPP4-i, and use of insulin would predict the occurrence of eGFR <60 mL/min. A statistically significant association was found with higher age (OR = 1.148; *P *< 0.001), presence of kidney stones (OR = 5.337; *P *= 0.002), absence of excess weight or obesity (OR = 3.829; *P* < 0.001), and use of insulin (OR = 2.482; *P *= 0.043). 

To explore the relationship between CKD and the presence of ACR >= 30 mg/g along with several variables (age, known DM duration, use of biguanide, and use of GLP1-a), a second model was constructed. However, only the use of GLP1-a showed statistical significance as a predictor (OR = 4.653; *P *= 0.042) of the presence of MA in the studied population.

## Discussion

The results obtained in this study provide important information about the impact of DM and its complications on the studied population. Data analysis demonstrated that most patients (284, 76.2%) had their DM controlled, an encouraging outcome highlighting the effectiveness of therapeutic interventions and medical follow-up in PCU. However, an association was observed between increased disease duration and poorer glycemic control, indicating the need for more effective strategies to manage DM in the advanced stages of the disease.

It was also noted that most studied patients had one or more comorbidities, with a high prevalence of excess weight or obesity (292, 81.8%) and HT (284, 79.6%). While it is recognized that both poor glycemic control and the presence of comorbidities increase the risk of target organ damage, it is important to consider that many of these injuries are subclinical and may go unnoticed due to a lack of obvious symptoms. Therefore, regular screening for target organ damage, such as MA, is crucial for the prevention of severe complications of DM.

CKD prevalence with eGFR < 60 mL/min/1.73 m^2^ was 24.9% (*n* = 89). A few recent studies adapted to the Portuguese population described the prevalence of CKD, with eGFR <60 mL/min in diabetic patients [9,10]. Nonetheless, a North American study showed that the prevalence of moderate to severe renal failure based on eGFR < 60 mL/min among patients with type 2 DM was 18% [11].

It is interesting to note that statistically significant differences were not found between the groups with eGFR equal to or greater than 60 mL/min and those with less than 60 mL/min regarding HbA1c levels, suggesting that other factors may be contributing to the progression of CKD, besides glycemic control. There is also the hypothesis that despite users having controlled values at the time of data collection, they may have experienced periods of poor control before and/or since diagnosis, leading to inherent irreversible renal function deterioration. Additionally, the absence of significant differences among the PCUs indicates a relative uniformity in the quality of care provided to diabetic patients.

The presence of ACR >= 30 mg/g, compatible with the presence of at least MA, observed in 68 (19%) patients, was an important marker of endothelial injury and a significant predictor of cardiovascular morbidity and mortality. Published data vary markedly (from 4% to 46%), not providing a concrete idea of the problem [12-17]. There is some prevalence data on MA in Portugal. In 1998, a study published in the journal of the Portuguese Society of Internal Medicine concluded that the prevalence of MA was around 25% in patients with HT, 13% in patients with type 1 DM, and 25% in patients with type 2 DM [9,10]. In 2007, a study with 1,582 hypertensive diabetic patients showed a positive test rate (with Micral‐Test®) for MA of 29% [18]. The discrepancy in the prevalence of MA in the various studies can be explained by the different characteristics of the studied populations and the methods used for screening.

In this study, ACR >= 30 mg/g was more common with longer known DM duration in individuals medicated with GLP1-a (*P *= 0.046). Conversely, it was less frequent in patients medicated with biguanides (*P *= 0.035). This can be explained by the fact that the presence of MA is associated with more advanced stages of the disease and, consequently, with patients requiring multiple therapeutic classes for glycemic control. On the other hand, biguanides are frequently used in the earlier stages of the disease. The association between ACR >= 30 mg/g and time since DM diagnosis, as well as the type of medication used, highlights the importance of continuous and personalized monitoring for patients at different stages of the disease.

When regarding the presence of both eGFR and/or ACR compatible with CKD, a total of 130 (36.4%) individuals were identified as having CKD. This means that approximately one in every three people with DM has CKD, with consequent implications for therapeutic management and a higher risk of end-stage renal disease, cardiovascular morbidity, and mortality. From these, only around a fifth had CKD correctly identified and codified, and most of these patients had other causes for CKD besides DM. This fact makes us think that renal deterioration due to DM tends to be undersighted, which highlights the importance of this study to better understand the current reality of this population and provide the best response to these patients.

Indeed, this study is a pioneer in its geographical area and can be crucial for improving the services provided by healthcare units. Additionally, the results obtained can assist in the decision-making of other healthcare units, offering valuable insights for the enhancement of care provided to DM patients in the region. However, it is important to note that the sample of patients is small and confined to a limited geographical area, which may hinder the generalization of this study to other units and even to the Portuguese reality.

Besides, we should also take into consideration that the Cockcroft-Gault modified formula has its limitations. Nonetheless, it is still widely used in clinical practice and recent articles and reviews continue to support it [19].

Despite the significant contributions that this study can offer, it is necessary to consider the local specificities and variations in the provision of healthcare in different geographical and institutional contexts. New, more comprehensive studies using glomerular filtration rates instead of estimates may help us better comprehend and adjust our practice to the current reality.

## Conclusions

In conclusion, this study sheds light on the complex interplay between DM and CKD, providing valuable insights into the prevalence, risk factors, and management strategies for these conditions within a primary healthcare setting. The findings underscore the substantial burden of renal impairment among diabetic patients, with approximately one-third exhibiting CKD based on eGFR and/or ACR criteria. The presence of at least MA, a marker of endothelial damage and cardiovascular risk, further underscores the importance of proactive screening and personalized management strategies for diabetic patients. The study's observation that ACR >= 30 mg/g prevalence correlates with disease duration and specific medication usage emphasizes the need for tailored interventions targeting different stages of DM progression. Moreover, the study's identification of CKD cases often going undiagnosed or misclassified underscores the imperative for improved recognition and coding of renal impairment in clinical practice. This underscores the importance of raising awareness among healthcare professionals regarding the significance of renal function assessment in diabetic patients and the need for accurate documentation to guide appropriate management.

Despite the study's limitations, including its small sample size and regional confinement, its pioneering nature in the geographical area and its contribution to understanding the local epidemiology of DM-associated CKD are noteworthy. Moving forward, further research efforts, particularly large-scale studies with broader geographical representation, will be instrumental in advancing our understanding and management of this significant public health challenge. Overall, this study serves as a call to action for healthcare providers to adopt a proactive and personalized approach to diabetes care, emphasizing early detection and comprehensive management to mitigate the burden of renal complications in diabetic patients.
